# CRISPR-based gene expression platform for precise regulation of bladder cancer

**DOI:** 10.1186/s11658-024-00569-7

**Published:** 2024-05-09

**Authors:** Tianying Zhan, Xiao Li, Jiumin Liu, Chujin Ye

**Affiliations:** 1 Department of Clinical Laboratory, Guangdong Provincial Key Laboratory of Major Obstetric Diseases; Guangdong Provincial Clinical Research Center for Obstetrics and Gynecology; The Third Affiliated Hospital of Guangzhou Medical University, Guangzhou, China; 2 Department of Urology, Guangdong Provincial People’s Hospital, Guangdong Academy of Medical Sciences, Guangzhou, China; 3 Guang Dong Medical Academic Exchange Center, Guangzhou, China; 4 Department of Urology, Carson International Cancer Centre, Shenzhen University General Hospital, Shenzhen, China

**Keywords:** CRISPR system, Bladder cancer, Gene regulation tool, Transgene, Gene therapy

## Abstract

The development of compact CRISPR systems has facilitated delivery but has concurrently reduced gene editing efficiency, thereby limiting the further utilization of CRISPR systems. Enhancing the efficiency of CRISPR systems poses a challenging task and holds significant implications for the advancement of biotechnology. In our work, we report a synthetic dual-antibody system that can stably exist in the intracellular environment, specifically inhibiting the functions of NF-κB and β-catenin. This not only elevates the transgenic expression of the CRISPR system by suppressing the innate immune response within cells to enhance the gene editing efficiency but also demonstrates a notable tumor inhibitory effect. Based on the specific output expression regulation of CRISPR-CasΦ, we constructed a CRISPR-based gene expression platform, which includes sensor modules for detecting intracellular β-catenin and NF-κB, as well as an SDA module to enhance overall efficiency. In vitro experiments revealed that the CRISPR-based gene expression platform exhibited superior CDK5 expression inhibition efficiency and specific cytotoxicity towards tumor cells. In vitro experiments, we found that CRISPR-based gene expression platforms can selectively kill bladder cancer cells through T cell-mediated cytotoxicity. Our design holds significant assistant potential of transgene therapy and may offer the capability to treat other diseases requiring transgene therapy.

**Supplementary Information:**

The online version contains supplementary material available at 10.1186/s11658-024-00569-7.

## Introduction

Bladder cancer ranks among the prevalent malignancies affecting the genitourinary system. Although the current therapy has the potential to decrease the recurrence rate of some patients to a certain extent, the effect is unsatisfactory due to poor specificity, great side effects and unknown potential mechanism of regulating tumor [1]. While certain clinical trials have showcased the promise of cancer immunotherapy, this method is constrained by significant challenges, notably the absence of specific antigens targeted exclusively to the tumor [2–4]. However, identifying surface antigens that are highly specific to tumors proves challenging, constraining the spectrum of targetable tumors and thereby impeding the effectiveness of immunotherapy.

Synthetic RNA-based switch is an increasingly common application in synthetic biology to engineer cell function, which involves incorporating aptamer domains that can sense small molecules into the regulation of gene expression of interest [5]. Aptamers show a number of advantages over traditional protein-based transcription controllers [6]. Although proper function of RNA switches depends on a large extent on optimal connections between aptamers and catalytic cores, regulatory modifications of expressed genes by these switches occupy little space of the genome. In addition, these modular designs make it easier to change genes of interest and do not require additional protein cofactors to function. Sensor units can be reprogrammed and incorporated into any RNA of interest to enable a variety of regulatory functions.

The promising prospects of targeted gene expression regulation in tumor therapy have been observed. However, the genetic elements involved in gene regulation, when introduced as exogenous substances, face the challenge of being targeted by the intracellular innate immune response (IIR), thereby diminishing the efficiency of gene expression [7, 8]. The IIR serves as the initial defense mechanism against infections of cells. It encompasses various enzymes and pathways designed to shield cells from harm caused by foreign DNAs [9]. Within mammalian cells, DNA is typically contained within the nucleus, while the presence of DNA in the cytoplasm is interpreted as a potential indicator that the cell is infected [9, 10]. Once IIR detects cytoplasmic DNAs, this event will trigger a cascade of changes in the cell’s cascade kinase, which inhibits the expression of the transgene [11, 12]. The ANAM system had been constructed to improve the efficiency of transgenic by inhibiting NF-κB and β-catenin, the key signaling molecules of IIR [13]. Inhibition of different parts of the IIR system can improve transgenic expression in mammalian cells to varying degrees.

In the present work, we had developed a double-specific antibody that binds with high affinity to endogenous β-catenin and NF-κB in cancer cells and inhibited their function. By inhibiting these key enzymes of IIR, the overall transgenic efficiency was improved. Moreover, a novel synthetic gene expression platform was designed to activate and induce T cells killing bladder cancer cells based on CRISPR-CasΦ specifically by identifying both endogenous β-catenin and NF-κB.

## Methods

### Cell lines used and cultured

HEK-293t (Cat. No: CVCL_B2R2) cells and cancer cells T24 (Cat. No: CVCL_0554), UMUC3 (Cat. No: CVCL_1783), and SW780 (Cat. No: CVCL_1728) were cultivated in DMEM (Gibco^®^, Life Technologies, Carlsbad, CA), supplemented with 5% fetal bovine serum (FBS) and 100 U/ml penicillin/streptomycin, under conditions of 37 °C and 5% CO_2_.

### Human primary T cells

We purchased human primary T cells from OriBiotech (Shanghai, China) and cultured them in RMPI1640 supplemented with 30 units/mL IL-2 (Beijing T&L Biotechnology CO, LTD, Beijing, China) and 5% fetal bovine serum (FBS). The T cells were amplified with Dynabeads^®^ Human T-Activator CD28/CD3 (Gibco^®^, 11163D). Dynabeads^®^ were added at a cell to bead ratio of 1:1. The beads were separated with a magnet after activation for 3 days, and the cells were cultured for at least one week before they were used for experiments.

### RNA extraction and real-time quantitative PCR

At 48 h post-transfection, total RNA was isolated from cells transfected with the plasmid using trizol reagent (Invitrogen). The RevertAid First Strand cDNA Synthesis Kit (Fermentas, Hanover, MD, USA) was employed for synthesizing cDNAs from the total RNA. Real-time quantitative PCR was carried out on an ABI PRISM 7000 Real-Time PCR System (Applied Biosystems, Foster City, CA, USA) using an All-in-One qPCR combination kit (GeneCopoeia, Rockville, MD, USA). The specific protocol was executed following the manufacturer’s instructions. The primers utilized in this study are detailed in the Additional file 1. Each experiment was replicated at least three times.

### Measurement of fluorescent protein expression

For the assessment of fluorescent protein expressions, the examined cells were suspended in DMEM and subjected to analysis utilizing an lsii Fortessa cytometer (BD Biosciences, San Jose, CA, USA). Subsequent data analysis was conducted using FlowJo software (TreeStar, Ashland, OR, USA). Each experiment was replicated a minimum of three times.

### Cell apoptosis assay

Following the manufacturer’s guidelines, the cell death detection ELISA method (Roche Applied Science, Penzberg, Germany) was employed for the quantitative detection of histologically bound DNA fragments (nucleosomes) within the cytoplasm to assess apoptosis. Absorbance, proportional to the quantity of nucleosomes released into the cytoplasm, was measured using a microplate reader (Bio-Rad) at a wavelength of 405 nm. The experiment was triplicated for each sample, and the entire procedure was reiterated at least three times.

### Statistical analysis

The data were presented as means ± standard deviation (SD). Statistical analyses were conducted using SPSS statistical software, version 20.0 (SPSS, Chicago, IL, USA). Significance tests were carried out utilizing Student’s t-tests or analysis of variance. A *p*-value less than 0.05 was considered statistically significant.

## Results

### The design and construction of the smart AND-gate gene circuit

The artificial gene circuit can realize the specific recognition and characteristic regulation of cells. Dual signal recognition of AND-gate gene circuits has been confirmed to identify cancer cells [14, 15]. However, the previous design only reprogramed the cancer cells in vitro. Adeno-associated viruses (AAV) stand out as promising vectors for gene therapy, while their utility is restricted by their limited carrying capacity. Only reasonable design of target gene expression system can AAV be loaded with multifunctional gene circuits [16].

**Fig. 1:**
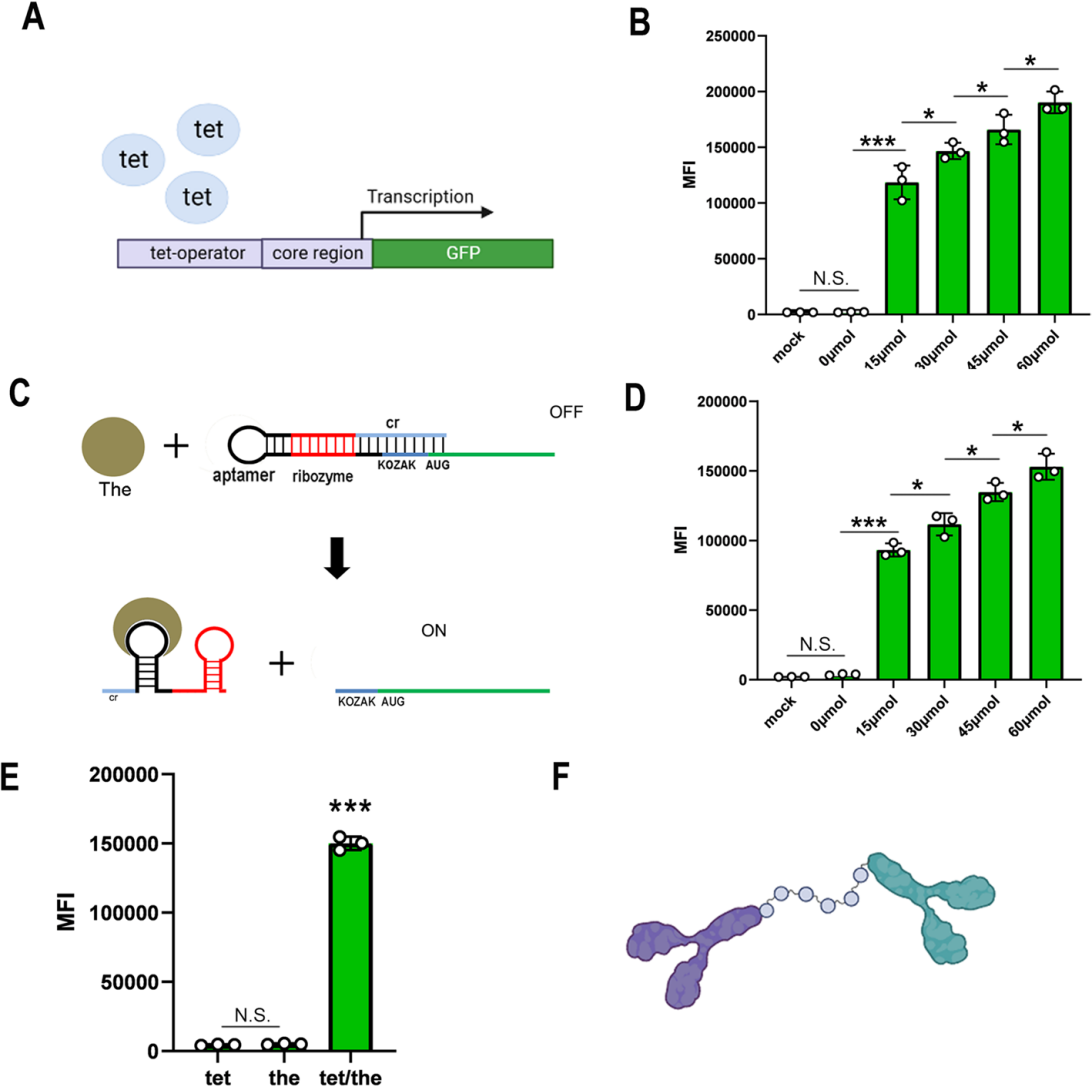
The effect of the promoter-sensor and aptazyme-sensor. **A** The structure diagram of the promoter-sensor sensing the endogenous tetracycline. **B** The promoter-sensor triggered the expression of GFP with different tetracycline concentrations (0, 15, 30, 45 and 60 μmol/L) in HEK293t cells. HEK293t cells transfected with the control vector were set as the mock group. **C** The structure diagram of the aptazyme-sensor sensing the endogenous theophylline. **D** The promoter-sensor triggered the expression of GFP with different theophylline concentrations (0, 15, 30, 45 and 60 μmol/L) in HEK293t cells. HEK293t cells transfected with the control vector were used as the mock group. **E** The mean relative fluorescence intensity (MFI) of GFP expression cells. **F** The structure diagram of the antibody system

The specific promoter and aptazyme-based mRNA were design to be the sensors recognizing different molecular signals in our work. First, tetracycline was used as a model signaling molecule to determine the effectiveness of the sensors. The specific promoter (promoter-sensor) sensing tetracycline were constructed and validated, and the strategy of promoter construction referred to previous study [17] (Fig. 1A). In the proof-of-concept experiment, different tetracycline concentrations were set and determined the tetracycline sensitivity of promoter for effectiveness and concentration dependence (Fig. 1B). Next, the mRNA transcribed by the gene of interest was redesigned using the aptazyme (aptazyme-sensor) of theophylline referred to previous work [18] (Fig. 1C). The effectiveness of the aptazyme-sensor was tested by the addition of theophylline molecules (Fig. 1D). We found that both promoter-sensor and aptazyme-sensor showed the ability to sense target signals and sensitive to activate in the expression of signaling molecules within cells. The promoter-sensor and aptazyme-sensor were combined to construct a novel synthetic gene expression platform (NSGEP) that driven the gene of interest expressing based on sensing two different signals at the same time. We found that the GFP could be expressed driven by the NSGEP only when both theophylline and theophylline were present in the HEK-293t cells (Fig. 1E).

### Effects of the specific double antibody on transgene expression

The ANAM system had been reported to be constructed to enhance expression of transgene by inhibiting endogenous β-catenin and NF-κB. However, single-stranded RNA molecules were unstable in the intracellular environment, which limited the further use of the ANAM system in enhancing expression of transgene [13]. To overcome this limitation, we redesigned and constructed a specific double antibody (SDA) that was more stable in the intracellular environment (Fig. 1F). Here, we developed and implemented the SDA system with the aim of suppressing the activities of NF-κB and β-catenin while enhancing transgene expression. Our findings revealed that both the ANAM system and the SDA system effectively inhibited the functions of β-catenin and NF-κB. Intriguingly, we observed that the antibody system exhibited a superior inhibitory effect on the downstream gene expression of β-catenin and NF-κB compared to the ANAM system.

**Fig. 2:**
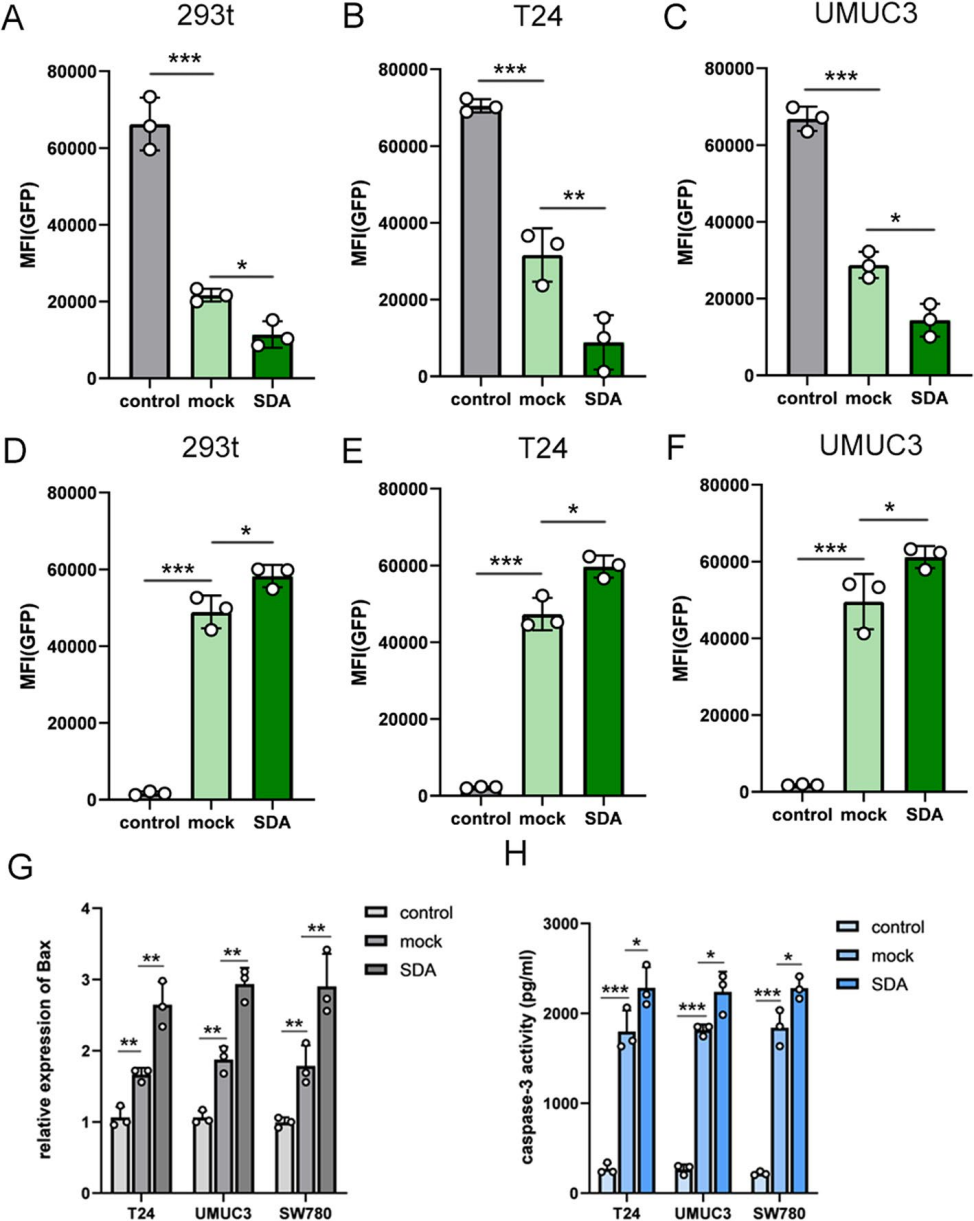
The efficiency of gene editing of CRISPR-CasΦ and its derivative system using the GFP reporter gene. The impact of DAS on enhancing the efficiency of CRISPR-CasΦ gene editing in 293t (**A**) T24 (**B**) and UMUC3 cells (**C**) cells was evaluated using flow cytometry. The impact of DAS on enhancing the efficiency of CRISPR-dCasΦ-VP64 gene editing in 293t (**D**) T24 (**E**) and UMUC3 cells (**F**) cells was evaluated using flow cytometry. The qPCR assays revealed that CRISPR-dCasΦ-VP64 successfully induced the expression of the Bax gene in T24 cells, UMUC3, and SW780 cells. **G** The ELISA assay results indicated that the expression of caspase-3 exhibited variation corresponding to the expression levels of the Bax gene in T24 cells, UMUC3, and SW780 cells (**H**). All experiments were repeated three times. *< 0.05, **< 0.01, ***< 0.001

Our goal was to enhance the gene editing efficiency of the CRISPR-CasΦ system which is carried with plasmids by using SDA to improve the expression of CRISPR-CasΦ [19]. We used SDA to enhance the ability of the CRISPR-CasΦ system on targeted DNA via inducing non-homologous end-joining. The GFP served as the targeted gene and was knocked down by the CRISPR-CasΦ system that transported using plasmids. As expected, in the group SDA-transfected cells, we observed that the degree of GFP inactivation was significantly higher than in the control groups. The expressions of GFP were quantified using flow cytometry analysis (Fig. 2A–C). In addition, we demonstrated that SDA can improve the gene-editing capability of CRISPR-dCasΦ that fused with other effector protein such as VP64 (Fig. 2D–F).

To further explore the applications of SDA in conjunction with CRISPR-CasΦ/ dCasΦ, we conducted in vitro experiments to assess its potential enhancement of gene editing efficiency on endogenous bax gene, a marker for cell apoptosis [20]. We up-regulated the expression of Bax using the CRISPR-dCasΦ-VP64 system. As expected, the CRISPR-dCasΦ-VP64 system successfully triggered the activation of the Bax gene expression (Fig. 2G), leading to the induction of apoptosis in cancer cells (Fig. 2H).

### Effects of the AND-gate gene circuit on triggering the immune killing response in bladder cancer cells in vitro

**Fig. 3:**
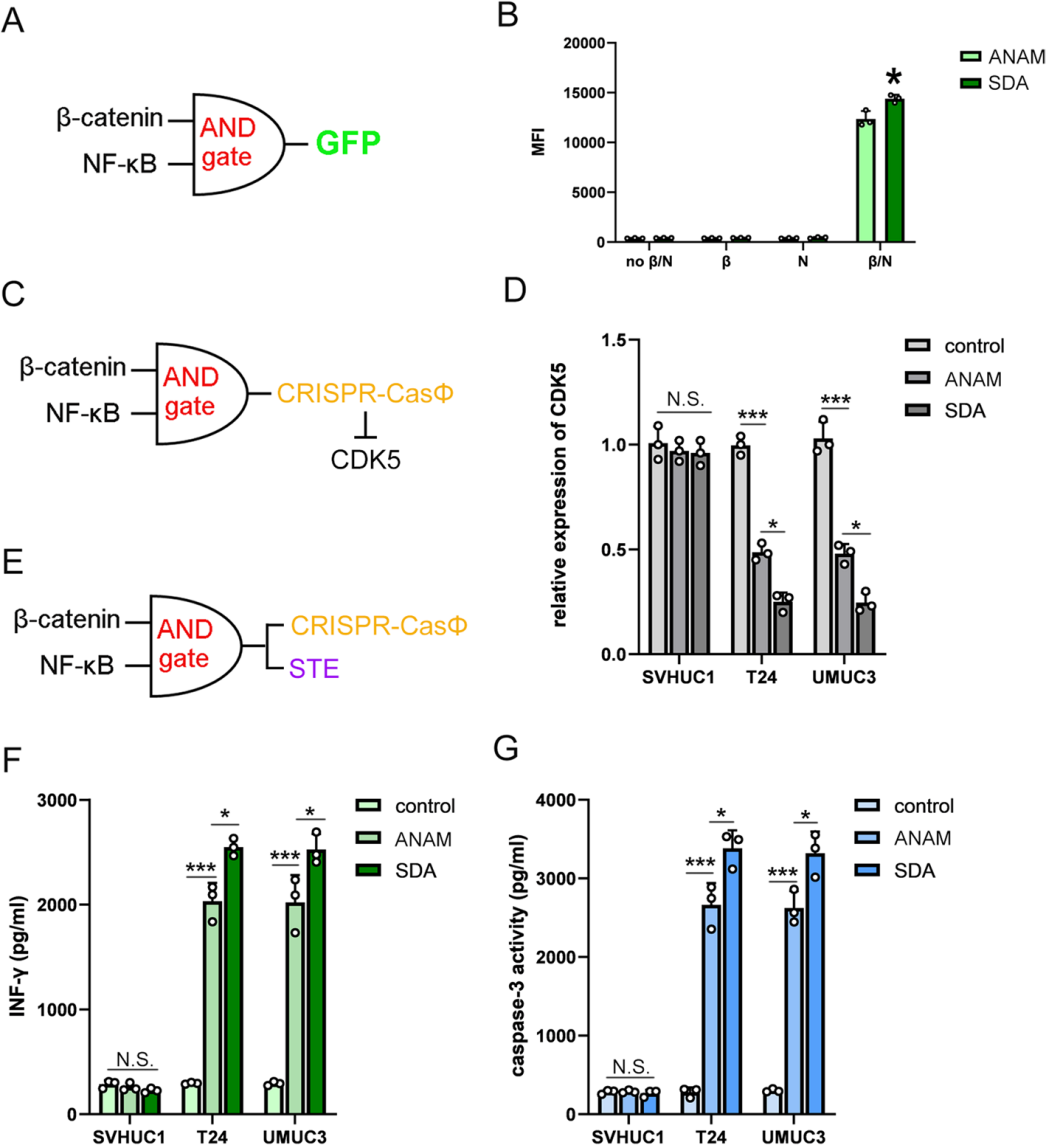
The effect of the AND-gate gene circuit killing cancer cells in vitro. **A** Structure diagram of the NSGEP driving the expression of GFP. **B** The β-catenin and NF-κB recognition based AND-gate gene circuit triggered the expression of GFP. **C** Structure diagram of the NSGEP driving the expression of the CRISPR-CasΦ on CDK5 expression. **D** Expression levels of CDK5 in SVHUC1, T24 and UMUC3. **E** Structure diagram of the NSGEP driving the expression of the CRISPR-CasΦ on CDK5 expression and STE. **F** The β-catenin and NF-κB recognition-based NSGEP induced the T cells to kill bladder cancer selectively. **G** ELISA was used to determine INF-γ expression in vitro. All experiments were repeated three times. *< 0.05, **< 0.01, ***< 0.001

To investigate whether a gene circuit could specifically treat cancer, we used the NSGEP based on recognition of β-catenin and NF-κB to drive the expression of therapeutic gene in bladder cancer cells specifically. The NSGEP was composed of the promoter-sensor that can specifically detect endogenous β-catenin as well as the aptazyme-sensor that specifically detect NF-κB (Fig. 3A). In the proof-of-concept experiment, the promoter-sensor sensing β-catenin was used to drive expression of the aptazyme-sensor sensing NF-κB. The expression of GFP was utilized to determine the effectiveness of the AND-gate circuit. We found that the expression of GFP was greatest only when both β-catenin and NF-κB were present (Fig. 3B). In other words, the NSGEP only worked effectively in the present of both β-catenin and NF-κB signals.

We used the NSGEP to the drive the expression of CRISPR-CasΦ knocking down the Cyclin-dependent kinase 5 (CDK5) gene in bladder cancer cell specifically, which would inhibited the expression of interference on regulatory factor 2 to down-regulate the expression level of PD-L1 on the surface of tumor cells [21]. The normal bladder mucosal epithelial cell line SVHUC1 and bladder cancer cell lines (T24, UMUC3) were used to determine the efficiency and specificity of CRISPR-CasΦ driven by NSGEP on knocking down CDK5 expression in bladder cancer cells (Fig. 3C). We found that CDK5 was inhibited only in T24 and UMUC3 cells, and the inhibitory effect of CDK5 was more obvious in the presence of SDA, indicating that NSGEP only driven the expression of CRISPR-CasΦ only in cancer cells and the SDA could improve the efficiency of CRISPR-CasΦ (Fig. 3D).

The AND-gate gene circuit was then used to drive expression of a surface T cell engagers anchored anti-CD3 STE, which induced T cells to kill cancer cells specifically [14] (Fig. 3E). The SDA was integrated into the gene circuit to maximize the efficiency of the entire expression system. T cells were amplified in vitro and co-cultured with cancer cells to determine the killing effect of T cells on cancer cells. The results showed that the β-catenin and NF-κB recognition based gene circuit worked in bladder cancer cells, and that expression of CD3-scFv driven by the gene circuit could induce T cells to kill tumor cells effectively. More importantly, the gene circuit integrated with antibody system expression had a stronger therapeutic effect (Fig. 3F). Next, the expression levels of INF-γ in the supernatant of T cells co-cultured with T24 cells were measured by ELISA to assess the level of T cell activation (Fig. 3G). We found that the NSGEP could not only activate T cells, and we also determined that the integrated antibody system expression system had higher transgene efficiency.

## Discussion

In our study, we demonstrated that gene circuits could produce immunomodulatory components with a high degree of specificity only within cancer cells. These circuits can be loaded into AAV and delivered directly from in vitro to in vivo and induce an effective antitumor response. Our modular AAV therapy system design strategy enables future clinical conversion. However, several important problems must be solved in the future clinical application of gene circuits. The design and validation of the gene circuit should be extended to include additional models, such as patient-derived tumor cells. The potential off-target activity of gene circuit components requires more rigorous testing. Consequently, future research should delve into assessing the specificity and potential side effects within the in vivo environment.

There is great potential for our therapeutic approach to be combined with other treatment strategies. T cells are attracted to the tumor site and activated by tumor-specific expression of immunomodulatory combinations. The destruction of the immunosuppressive microenvironment is one of the ways to enhance the efficacy of engineered T cells. Furthermore, recent studies have highlighted that immunogenic neoantigens, susceptible to targeting by the host immune system, may be expressed on the surface of tumor cells. We propose the hypothesis that using our gene circuit strategy in combination with neoantigen-based immunotherapy to trigger tumor killing may be an effective research approach. For example, our gene circuit can specifically express neoantigens in tumor cells. In addition, the expression of secreted immune-modulators from cancer cells enhances neoantigen presentation by antigen-presenting cells, which further enhances the antitumor immune response.

Because our study was able to specifically express therapeutic elements in tumors, it may provide a fundamental framework for studying cancer biology. In addition, synthetic gene circuits have demonstrated the capability to detect disease markers beyond the confines of the cell. Our strategy for sensing cell states using an artificial promoter combination can be adapted for addressing other intricate diseases. Such diseases necessitate highly specific and multifactorial immune functional programming, achievable through straightforward modifications to the input and output of the gene circuit.

## Conclusions

In conclusion, the modulation of the immune system stands out as a promising approach for treating complex diseases. However, the mechanisms underlying disease are complex, and the effectiveness of a single therapy alone cannot meet the requirements for disease treatment. Overcoming this challenge can be achieved through the development of targeted immune combinations. Given the broad impact of the immune system on human physiology, it is crucial to restrict the expression of immune combinations to specific diseases. This approach is essential to prevent undesirable side effects and enhance efficacy. Therefore, highly specific, regulated and easily modified synthetic gene circuits will provide a promising approach for disease treatment.

### Supplementary Information

**Additional file 1. Table S1:** The sequences of ANAMs in this study. **Table S2:** Sequence of primers used in this study. **Table S3:** Sequence of AON-promoter.

## Data Availability

All data and materials used in this study are available upon reasonable request from the corresponding author.

## Abbreviations

CAR: Chimeric antigen receptor
IIR: Innate immune response
NSGEP: Novel synthetic gene expression platform
SDA: Specific double antibody
AON promoter: All-or-nothing promoter
CRISPR-CasΦ: Clustered regularly interspaced short palindromic repeats/CasΦ nuclease
ANAM: Artificial nucleic acid molecule
CDK5: Cyclin-dependent kinase 5
STE: Surface T cell engagers
GOI: Gene of interest
