# Supplementary material for: CRISPR-based gene expression platform for precise regulation of bladder cancer
Source: Cell Mol Biol Lett. 2024 May 9;29:66. doi: 10.1186/s11658-024-00569-7 (PMC11080256; doi:10.1186/s11658-024-00569-7)
Supplement: Supplementary file 1 — Additional file 1. Table S1: The sequences of ANAMs in this study. Table S2: Sequence of primers used in this study. Table S3: Sequence of AON-promoter. [file 11658_2024_569_MOESM1_ESM.docx]

Supplementary table 1: The sequences of ANAMs in this study

| name | relative sequences (5′-3′) |
| --- | --- |
| ANAM-β-κB | GGGAGCGGCCGATCTATGGACGCTATAGGCACACCGGATACTTTAACGATTGGCCGCCTGCTGCGGCATCCTGAAACTGTTTTAAGGTTGGCCGATGCCGCAGCAGTTCCC |

Supplementary table 2: Sequence of primers used in this study

| name | relative sequences (5′-3′) |
| --- | --- |
| MYC | F: GGCTCCTGGCAAAAGGTCA  R: CTGCGTAGTTGTGCTGATGT |
| Cyclin D1 | F: GCTGCGAAGTGGAAACCATC  R: CCTCCTTCTGCACACATTTGAA |
| TRAF1 | F: TCCTGTGGAAGATCACCAATGT  R: GCAGGCACAACTTGTAGCC |
| BclXL | F: GAGCTGGTGGTTGACTTTCTC  R: TCCATCTCCGATTCAGTCCCT |
| IFN-β | F: ATGACCAACAAGTGTCTCCTCC  R: GGAATCCAAGCAAGTTGTAGCTC |
| TNF-α | F: CCTCTCTCTAATCAGCCCTCTG  R: GAGGACCTGGGAGTAGATGAG |
| IL-12 | F: ACTCACCTCTTCAGAACGAATTG  R: CCATCTTTGGAAGGTTCAGGTTG |
| Bax | F: CCCGAGAGGTCTTTTTCCGAG  R: CCAGCCCATGATGGTTCTGAT |
| Bcl-2 | F: GGTGGGGTCATGTGTGTGG  R: CGGTTCAGGTACTCAGTCATCC |

Supplementary table 3: Sequence of AON-promoter

| name | relative sequences (5′-3′) |
| --- | --- |
| AON-promoter | CGCACTTTGCTGCCTCACGCGC**TATATAA**GCCAGCTTTGACCGCGTACGAACGAGATCGC |
